# Role of Toll-Like Receptor 4 on Osteoblast Metabolism and Function

**DOI:** 10.3389/fphys.2018.00504

**Published:** 2018-05-08

**Authors:** Ana Alonso-Pérez, Eloi Franco-Trepat, María Guillán-Fresco, Alberto Jorge-Mora, Verónica López, Jesús Pino, Oreste Gualillo, Rodolfo Gómez

**Affiliations:** ^1^Musculoskeletal Pathology Group, Laboratory 18, Institute IDIS, Servicio Galego de Saúde, Santiago de Compostela, Spain; ^2^Division of Traumatology, Santiago University Clinical Hospital, Santiago de Compostela, Spain; ^3^NEIRID LAB, Laboratory 9, Institute IDIS, Servicio Galego de Saúde, Santiago de Compostela, Spain

**Keywords:** osteoblast, TLR4, inflammation, LPS, osteoclast, MSCs, osteoclastogenesis, bone resorption

## Abstract

Inflammation is a process whose main function is to fight against invading pathogens or foreign agents. Nonetheless, it is widely accepted that inflammation takes part in multiple processes in a physiological or pathophysiological context. Among these processes the inflammation has been closely related to bone metabolism. It is well-known that in systemic inflammatory diseases such as rheumatoid arthritis the inflammatory environment contributes to the reduction of the bone mineral density. This has been further evidenced in different animals models of osteoporosis where the deletion of key inflammatory molecules dramatically reduced the bone loss. On the contrary, it is also well-known that certain degree of inflammation is required to allow bone fractures healing. In fact, excessive use of anti-inflammatory drugs inhibits bone fracture consolidation. The innate immune responses (IIRs) contribute to the development and maintenance of the inflammation. These responses have been observed in cells of the musculoskeletal system. Chondrocytes and osteoblasts are equipped with the molecular repertoire necessary to setting up these IIR, including the expression of several toll-like receptors. Specifically, toll-like receptor 4 (TLR4) activation in mesenchymal stem cells, osteoblasts, and osteocytes has been involved in catabolic and anabolic process. Accordingly, in this review we have summarized the current knowledge about the physiology of TLR4, including its signaling, and its endogenous agonists. In addition we have focused on its role on osteoblast metabolism and function.

## Introduction

Bone is a mineralized connective tissue that exerts important functions. It provides rigidity to de body, protects soft tissues, and contributes to the locomotion. Moreover, it stores calcium and phosphate harboring also the bone marrow (Datta et al., 2008). Bone is a dynamic tissue continuously being formed and resorbed (bone remodeling). This process is required to maintain the structural integrity of the skeleton allowing the repair of damaged tissue as well as the homeostasis of calcium and phosphorous metabolism (Teitelbaum, 2000). The adequate balance between bone destruction and bone formation determinates the correct bone metabolism (Velasco and Riancho, 2008).

Bone tissue contains multiple types of cells including osteoblasts, osteoclasts, osteocytes, immune cells, adipocytes, stem cells, etc. (Florencio-Silva et al., 2015). In contrast to osteoclasts, responsible for bone resorption, osteoblasts are the only cells in charge of bone formation; they synthesize almost all of the constituents of the bone matrix and regulate its mineralization. Once the bone matrix is completely formed, osteoblasts differentiate into osteocytes, which play major roles in the regulation of calcium homeostasis and bone remodeling (Ralston and Helfrich, 2012). Osteoblasts and osteoclasts, along with osteocytes form the bone-remodeling unit (Rosen and Bouxsein, 2006).

Inflammation has been closely related to bone metabolism (Claes et al., 2012). It is well-known that in systemic inflammatory diseases such as rheumatoid arthritis, pancreatitis, and others the inflammatory environment contributes to the reduction of bone mineral density and, therefore, to the development of osteoporosis (Hardy and Cooper, 2009; Haas et al., 2015). It is widely accepted that an excessive amount of pro-inflammatory cytokines in these pathologies promotes osteoclastogenesis (Hardy and Cooper, 2009). The increased osteoclastogenesis in turn involves the imbalance between bone formation and bone resorption (Hardy and Cooper, 2009). Nonetheless, the link between an altered bone metabolism and inflammation is not limited to the systemic inflammatory diseases. In fact, this relationship has also been observed in certain metabolic diseases such as obesity (Cao, 2011) and type II diabetes mellitus (Alblowi et al., 2009). The connection between inflammation and bone metabolism has been further evidenced in different animal models of osteoporosis where the deletion of the receptor for key inflammatory cytokines, like interleukin-1 (IL1) and tumor necrosis factor (TNFα), dramatically reduced the bone loss (Vargas et al., 1996). On the contrary, it is also well-known that certain degree of inflammation is required to allow bone fractures healing (Cottrell and O’Connor, 2010; Claes et al., 2012). In fact, excessive use of anti-inflammatory drugs inhibits bone fracture consolidation (Cottrell and O’Connor, 2010). As a result, a fine regulation of the inflammatory environment is required to preserve bone homeostasis and bone regenerative properties (Claes et al., 2012).

The innate immune responses (IIRs) contribute to the development and maintenance of inflammation. These responses are tightly regulated by the toll-like receptor (TLRs) family. Among these receptors toll-like receptor 4 (TLR4) stands out. This receptor is expressed in the musculoskeletal system where it plays a key role in the regulation of the inflammatory environment (Gómez et al., 2015). Accordingly, in this review we have summarized the current knowledge about the physiology of TLR4, including its signaling, and its endogenous agonists. In addition, we have focused on its role on osteoblast metabolism, viability, inflammatory responses, and function.

## Toll Like Receptors

Human body is constantly defending itself from highly changing pathogens, and other different harmful agents. The innate immune system has evolved in this environment selecting the adaptability as an essential feature. This condition has involved that, in order to improve its efficiency, the immune system recognizes diverse biological patterns conserved across multiple pathogens rather than specific molecules. These patterns are known as pathogens-associated molecular patterns (PAMPs) (Janeway, 2013).

The receptors that recognize these structures are called pattern-recognition receptors (PRRs). They take part in the first and non-specific response of the immune system. We can find them at three different locations, secreted at the extracellular space, in the cytoplasmic membrane, or as intracellular molecules. For example, lipopolysaccharide (LPS)-binding protein, or C-reactive protein (CRP) are secreted PRRs, and nucleotide-binding oligomerization domain (NOD)-like receptors (NLRs) are intracellular PRRs (Feerick and McKernan, 2017). Instead TLRs, depending on its type and state of activation, can be found in the cytoplasmic membrane or at intracellular endosomes membranes (Chen and DiPietro, 2017). Despite these different locations the PRRs maintain structural and functional similarities, and they are highly conserved receptors.

**FIGURE 1:**
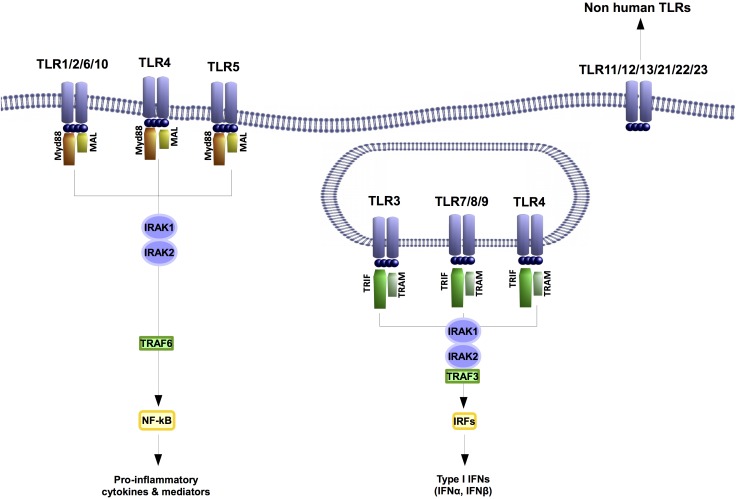
Toll like receptors are highly conserved proteins. In humans ten different TLRs have been described. TLR1, 2, 4, 6, and 10 are transmembrane receptors. The TLRs 3, 4, 7, 8, and 9 are located at endosomes. TLR4 is the only TLR that has been found at both transmembrane and endosome locations. Despite their localization all TLRs signaling through IRAK 1 and 2 kinases. Nonetheless, the outputs of their signaling will involve the expression of a different set of genes.

Toll-like receptors-mediated immune responses control inflammation-related catabolism including cell de-differentiation, induction of matrix metalloproteinases (MMPs) or inhibition of the expression of certain structural proteins. These receptors recognize PAMPs but some of them are also sense by damage-associated molecular patterns (DAMPs). These are host-derived molecules generated by damaged tissues. Among the 10 different TLRs described in humans so far, TLR4 is the TLR that detects more DAMPS (**Figure 1**). This precise TLR is the main PRR for LPS, but it is also activated by DAMPS related with different musculoskeletal pathologies like rheumatoid arthritis (RA) or osteoarthritis (OA) (i.e., Chen et al., 2007; Abdollahi-Roodsaz et al., 2011; Goldring and Scanzello, 2012).

## TLR4

TLR4 main expression is in myeloid origin cells like monocytes, macrophages, granulocytes, and also in the spleen (Medzhitov et al., 1997; Vaure and Liu, 2014). However, these are not the only cells where it is expressed, intestinal epithelium cells, brain endothelium cells, and adipocytes are able to react against LPS through TLR4 signaling (Vaure and Liu, 2014). Interestingly, it is noteworthy that chondrocytes (Wang et al., 2011), osteoblasts (Kikuchi et al., 2001) and synoviocytes (Midwood et al., 2009) express TLR4 receptor as well. This, along with the fact that TLR4 detects DAMPs, frequently associated with musculoskeletal pathologies, have supported the implication of this TLR in the pathophysiology of the musculoskeletal system (Midwood et al., 2009; Wang et al., 2011). Specifically, TLR4 has been linked to diseases like rheumatoid arthritis, osteoarthritis, and osteoporosis, where bone metabolism is altered (Abdollahi-Roodsaz et al., 2007; Kim et al., 2009; Gómez et al., 2015). Modulation or inhibition of TLR4 has been suggested as a treatment for these diseases. For instance, the inhibition of TLR4 in rheumatoid arthritis animal models, characterized by local and systemic reduction in bone mineral density, suppressed the severity of the disease (Abdollahi-Roodsaz et al., 2007). Moreover, TLR4 targeting has also been recently proposed as a potential treatment for osteoarthritis (Gómez et al., 2015), which is associated with subchondral bone alterations including osteophytes formation (bone spurs). In addition, in animal models of osteoporosis the pharmacological inhibition of its progression was also associated with an inhibition of TLR4 signaling (Vijayan et al., 2014).

Human TLR4 was the first characterized among all the mammalian TLRs (Medzhitov et al., 1997). This type I transmembrane protein is formed by 839 aminoacids, and is encoded by the gene located on chromosome 9q32-q33 (Keshava Prasad et al., 2009). TLR4 spans the cytoplasmic membrane. In contrast, the nucleic-acid-binding TLRs (TLR9, TLR8, TLR7, and TLR3) are confined to the membrane of intracellular endosomes (Chaturvedi and Pierce, 2009; Gangloff, 2012). TLR4, like all the TLRs and IL1 receptor (IL1R), are equipped with the same Toll/IL-1 receptor (TIR) domain (Keshava Prasad et al., 2009). Besides this, TLR4 has an extracellular leucine-rich repeat domain (Keshava Prasad et al., 2009). TLR4 has specific sites that have been associated with its activation and cellular localization through post-translational glycosylation and phosphorylation. Some of these modifications are essential for the correct function of this receptor. For instance, its glycosylation at Asn526 and Asn575 are vital for the expression of TLR4 on the cell surface. Likewise, the response of human cell lines to the PAMP LPS is blocked by the absence of two or more N-glycosylation sites in the TLR4 ectodomain (da Silva Correia and Ulevitch, 2002). TLR4 modifications are not limited to its extracellular domain since they also occur in the intracellular TIR domain (Medvedev et al., 2007; Raijmakers et al., 2010). In fact, in human cells phosphorylation of TLR4 at Tyr674 and Tyr680 are crucial for the correct signal transduction of this receptor (Medvedev et al., 2007).

## TLR4 Signaling

**FIGURE 2:**
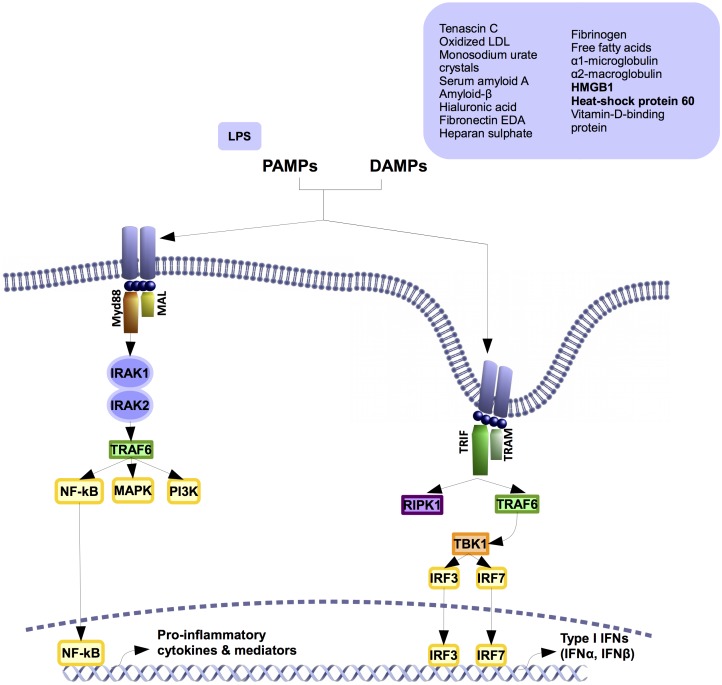
TLR4 signaling pathway. TLR4 activates two different pathways. Both of them share the co-factor (TIR)-domain-containing adapter protein (TIRAP, also known as MAL). In myeloid factor 88 (Myd88)-dependent signaling pathway, Myd88 polymerizes with IL1-receptor-associated kinases (IRAK) 1 and 2, inducing the activation of TNF-receptor-associated factor 6 (TRAF6). This activation boosts the signaling of different kinases like phosphatidylinositol-4,5-biphosphate 3 kinases (PI3K) and mitogen-activated- protein kinases (MAPKs), as well as the activation and transnucleation of Nuclear Factor kappa B (NF-κB). This leads to the production of pro-inflammatory cytokines and inflammatory mediators. Myd88-independent signaling pathway rather than recruit Myd88 it recruits TIR-domain-containing adapter protein inducing interferon-beta (TRIF) and TRIF-related adapter molecule (TRAM). The recruitment activates proteins like TNF-receptor-associated factor 6 (TRAF6), receptor-interacting serine/threonine-protein kinase 1 (RIPK1), and secondarily, TANK binding kinase 1 (TBK1). This elicits the transnucleation of interferon-regulatory factor (IRF) 3 and 7 transcription factors that leads to the production of different inflammatory factors like type I interferons.

In contrasts to other TLRs, TLR4 activates two different signaling pathways after its dimerization. The myeloid factor 88 (MyD88)-dependent and independent pathways (**Figure 2**). The canonical pathway, or MyD88-dependent pathway, is shared with the IL1R and all the TLRs. It later activates the alternative pathway or MyD88 independent pathway.

MyD88-dependent signaling pathway is initiated in the extracellular space. The activation starts by the recruiting of several co-factors including TIR-domain-containing adapter protein (TIRAP, also known as MAL) and MyD88 (Motshwene et al., 2009; Lin et al., 2010). Once this pathway is activated, MyD88 polymerizes and interacts with the intracellular IL1-receptor-associated kinases (IRAKs) 1 and 2 (Motshwene et al., 2009; Lin et al., 2010). The auto-phosphorylation of the IRAKs and the activation of TNF-receptor-associated factor 6 (TRAF6) (Medzhitov, 2001) triggers the signaling of phosphatidylinositol-4,5-biphosphate 3 kinases (PI3Ks), mitogen-activated- protein kinases (MAPKs), and the key pro-inflammatory transcription factor nuclear factor kappa B (NFκB) (Medzhitov, 2001). NFκB activates and leads to the induction of pro-inflammatory cytokines and pro-inflammatory mediators through the increased expression of certain enzymes such as the inducible nitric oxide synthase (iNOS) and the cyclooxygenase-2 (COX2) (Goldring and Goldring, 2007). Afterward, the receptor is internalized and activates the second pathway (Tanimura et al., 2008; Gangloff, 2012).

MyD88-independent signaling pathway takes place after MyD88-dependent pathway activation due to TLR4 internalization (Gangloff, 2012). This pathway involves the adapters TIR-domain-containing adapter protein inducing interferon-beta (TRIF) and TRIF-related adapter molecule (TRAM) (Kagan et al., 2008). The recruitment of TRIF by TRAM switches on the signaling proteins TRAF6, receptor-interacting serine/threonine-protein kinase 1 (RIPK1) and TANK binding kinase 1 (TBK1) (Gangloff, 2012). The activation of this pathway activates different transcription factors including NFκB, interferon-regulatory factor 3 (IRF3), interferon-regulatory factor 7 (IRF7), and their associated gene expression signature, which are mainly characterized by type I interferon gene expression (Gangloff, 2012). This alternative pathway is TLR4 and TLR3 specific (Kagan et al., 2008; Tanimura et al., 2008; Zanoni et al., 2011).

## TLR4 Agonists

Apart from LPS, TLR4 binds to multiple and diverse PAMPs (Chen et al., 2007) and DAMPs (**Figure 2**). These DAMPS include tenascin C (Midwood et al., 2009), oxidized LDL (Stewart et al., 2010), monosodium urate crystals (Scott et al., 2006), serum amyloid A (de Seny et al., 2013), amyloid-β (Stewart et al., 2010), hyaluronic acid (Liu-Bryan and Terkeltaub, 2010), fibronectin EDA (Chen et al., 2007), heparan sulfate (Chen et al., 2007), fibrinogen (Chen et al., 2007), some free fatty acids as lauric acid (Lee et al., 2004; Frommer et al., 2013), α1 microglobulin (Sohn et al., 2012), α2 macroglobulin (Sohn et al., 2012), high mobility group protein B1 (HMGB1) (Liu-Bryan and Terkeltaub, 2010; Yang et al., 2010), heat shock protein 60 (HSP60) (Kim et al., 2009) (Pevsner-Fischer et al., 2007), and vitamin-D-binding protein (Sohn et al., 2012). There also are some drugs that activate TLR4 such as opioids (morphine and oxycodone), or buprenorphine (Hutchinson et al., 2010).

Though the mechanisms to explain TLR4 promiscuity are not known, some hypotheses have been posed (Gómez et al., 2015). For instance, heterodimers formed with other TLRs (Stewart et al., 2010), as well as TLR4 interaction with several co-receptors and accessory molecules could be an explanation of the wide range of molecules recognized by this receptor (Scott et al., 2006; Stewart et al., 2010; Tsukamoto et al., 2010). MD-2, CD14, CD36 are some of these co-receptors that increase responsiveness to antigens or the wiliness to form favorable heterodimers (Scott et al., 2006; Stewart et al., 2010; Tsukamoto et al., 2010). One example is the fact that the CD36 mediated the formation of the heterodimer TLR4-TLR6 that bound oxidized LDL (Stewart et al., 2010). However, TLR4 homodimer could not recognize oxidized LDL as an agonist (Stewart et al., 2010). Notwithstanding, not every accessory molecules and co-receptors are indispensable. Some of them only improve DAMPs and PAMPs recognition, or the velocity of the signaling (da Silva Correia, 2001; Tsukamoto et al., 2010).

Sometimes certain DAMPs preparations used for research can be contaminated by some low-level of endotoxin (Tsan and Gao, 2007). This contamination could induce the activation of TLR4. Nevertheless, there are a pool of studies carried out in musculoskeletal tissues that include endotoxin contamination determination, and evidence how TLR4 is involved in the recognition of many DAMPs (Okamura et al., 2001; Pevsner-Fischer et al., 2007; Schelbergen et al., 2012; Sohn et al., 2012; de Seny et al., 2013; Frommer et al., 2013).

## TLR4 Expression in the Bone Compartment

TLR4 is highly expressed in human bone marrow (Uhlén et al., 2015). This is in line with the fact that TLR4 is highly expressed by immune cells (Shi et al., 2006), but also with the expression of TLR4 in adipocytes (Shi et al., 2006) and osteoblasts that also are present in the bone marrow (Kikuchi et al., 2001; Gasper et al., 2002; Zou et al., 2003; Nemoto et al., 2008). Human and mouse mesenchymal stem cells (MSCs), which are osteoblasts and adipocytes precursor cells, also express the mRNA of TLR4 (Pevsner-Fischer et al., 2007; Mo et al., 2008; Tomic et al., 2011; Chen et al., 2014; Huang et al., 2014; Albiero et al., 2015). Specifically, in human MSCs TLR4 expression was found induced along their osteoblastic differentiation (Mo et al., 2008).

Mesenchymal stem cells have been proposed as a tool to help in bone regeneration. MSCs used to this purpose can be obtained from different sources like bone marrow, adipose tissue, dental pulp, dental follicle, periodontal ligament, Wharton’s Jelly, or umbilical cord blood (UCB). According to this, it is noteworthy that the pattern of expression and activation of TLR4 is different across all these MSCs. In fact, TLR4 expression and activation in UCB-derived MSCs was found different from the one exhibited by MSCs from the bone marrow (van den Berk et al., 2009). Dissimilar expression of TLR4 was also observed in dental pulp MSCs in comparison to dental follicle MSCs (Tomic et al., 2011). This divergent expression correlated with different outcomes upon TLR4 activation like the diverse production of the transforming growth factor beta (TGF-β) (Tomic et al., 2011).

Interestingly, long-term activation of TLR4 in human bone marrow MSCs was related to a reduction in its expression (Mo et al., 2008). On the other hand in human bone marrow MSCs TLR4 activation by the DAMP HSP60 up-regulated TLR4 expression (Kim et al., 2009). This was also observed in mouse MSCs where TLR4 activation also up-regulated its own expression (Huang et al., 2014). Moreover TLR4 activation in the mouse osteoblastic cell line MC3T3-E1 induced its own expression (Liu et al., 2016). These apparent divergent results regarding TLR4 expression upon its stimulation might be explained according to different incubation times, agonist concentration, as well as cell-specific TLR4 expression rates.

## TLR4 and Osteoblast-Mediated Inflammation

**FIGURE 3:**
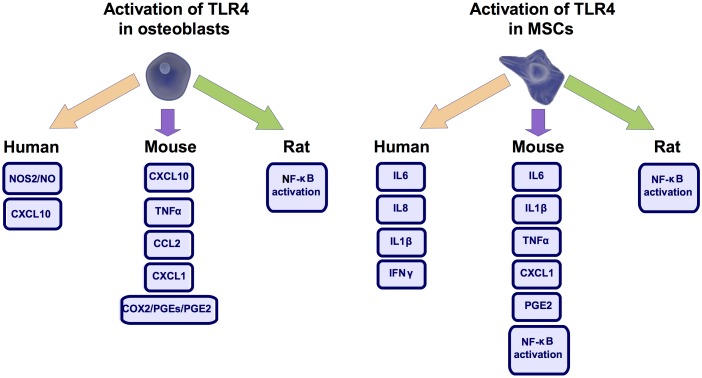
TLR4 mediated inflammation. Activation of toll-like receptor 4 (TLR4) in osteoblast and mesenchymal stem cells (MSCs) produce similar pro-inflammatory responses across different species. Nitric Oxide Synthase 2 (NOS2), also know as iNOS, C-X-C motif chemokine ligand 10 (CXCL10), Cyclooxygenase 2 (COX2), Prostaglandin-E (PGE), Prostaglandin-E2 (PGE2), Tumor necrosis factor (TNFα), C-C motif chemokine-ligand 2 (CCL2), C-X-C motif chemokine ligand 1 (CXCL1), Nuclear Factor kappa B (NF-κB), Interleukin-6 (IL6), Interleukin-8 (IL8), Interleukin-1β (IL1β), Interferon γ (IFNγ).

Excessive inflammation has been associated to bone loss (Inada et al., 2006). Interestingly, despite the structural role often depicted for osteoblasts they are also capable of mounting an inflammatory response (Gasper et al., 2002). TLR4 plays a key role in this activity (**Figure 3**). It was observed in human and mouse osteoblasts that TLR4 activation up-regulated the expression of C-X-C motif chemokine ligand 10 (CXCL10) (Gasper et al., 2002; Nakao et al., 2013), a known chemotactic factor (Gasper et al., 2002). TLR4 activation in human osteoblast cells also induced iNOS activity and nitric oxide production (Sosroseno et al., 2009), a known inflammatory mediator (Wu et al., 2007). This effect required the signaling through protein kinase A (PKA), PKC, phospholipase A2 (PLA2), lypoxygenase, tyrosine kinase activity, and the accumulation of cAMP (Sosroseno et al., 2009). TLR4 activation also increased in mouse osteoblasts the expression of COX2 and membrane-bound prostaglandin E synthase 1 (mPGES-1) as well as the production of TNFα (Zou et al., 2003), C-C motif chemokine-ligand 2 (CCL2) (Nakao et al., 2013), C-X-C motif chemokine ligand 1 (CXCL1) (Nakao et al., 2013), and prostaglandin E 2 (PGE2) (Inada et al., 2006). Regarding this, it was determined in the femur of mice defective for mPGES-1 that PGE2 production was required for the TLR4-mediated bone loss (Inada et al., 2006). In line with these TLR4-mediated pro-inflammatory activities, it was described in primary rat osteoblasts that TLR4 activation by the DAMP HMGB1 promoted the nuclear translocation of NFκB (Li et al., 2016), a key transcription factor involved in inflammation development (Clancy et al., 2004; Wen et al., 2006). Nonetheless, in the mouse osteoblastic cell line MC3T3-E1 this effect of HMGB1 was not attributed to TLR4 activation (Qiu et al., 2016). Despite this and underpinning the inflammatory role proposed for TLR4 in osteoblasts, it was observed in mouse osteoblasts that the inhibition of the formation of the TLR4-MyD88 complex significantly blunted the inflammatory responses elicited by this receptor (Nakao et al., 2013).

As observed in osteoblasts, in mouse MSCs TLR4 activation by PAMPs or DAMPs induced the secretion of IL6 (Pevsner-Fischer et al., 2007; Huang et al., 2014; He et al., 2015), IL1β (Huang et al., 2014; He et al., 2015), TNFα (Huang et al., 2014), as well as the nuclear translocation of NFκB (Pevsner-Fischer et al., 2007; Huang et al., 2014). Similar results were observed in human periodontal ligament MSCs where TLR4 activation increased the expression of IL6 and IL8 mRNAs (Albiero et al., 2015). Likewise, in human UCB MSCs TLR4 activation induced IL1β (Zhang et al., 2015), Interferon γ (INFγ) (Zhang et al., 2015), IL6 (van den Berk et al., 2009) and IL8 production (van den Berk et al., 2009).

## TLR4 Effect on Cell Viability and Proliferation

TLR4 activation, apart from its associated inflammatory effects, it has also been involved in the regulation of cell viability (Kim et al., 2009). Specifically, in human osteoblast-lineage cells it induced the caspase-dependent intrinsic apoptotic pathway, as well as the activation of p38 kinase and NFκB (Kim et al., 2009). Consistent with this TLR4 activation mediated the anti-citrullinated protein antibodies (ACPA) induced apoptosis of human osteosarcoma cells (SaOs-2 cell line) (Lu et al., 2016). However other reports revealed that TLR4 activation enhanced mouse osteoblast precursor cells proliferation (MSCs) (Wang et al., 2009; Huang et al., 2014; He et al., 2015) and protected them from oxidative stress-induced apoptosis, through a PI3K/Akt dependent mechanism (Wang et al., 2009). In contrast, in mouse MSCs TLR4 activation did not affect their proliferation or apoptosis rate (Chen et al., 2014). In the same way, in primary rat osteoblasts as well as in primary fetal rat calvaria osteoblasts TLR4 activation did not affect their proliferation rate or viability (Kadono et al., 1999; Li et al., 2016). Moreover, a recent report determined that TLR4 activation had no effect on the viability of mouse MSCs (He et al., 2015) as well as in MSCs derived from human periodontal ligament (Albiero et al., 2015).

All these studies depict an apparent contradictory scenario. However, the majority of reports performed with TLR4 agonists (DAMPs or PAMPs) on different osteoblastic lineage cells (MSCs and osteoblasts) revealed no alterations on cell viability (apoptosis or non-programmed cell death) (Kadono et al., 1999; Huang et al., 2014; Albiero et al., 2015; He et al., 2015; Li et al., 2016). These results were consistent across diverse species.

Nonetheless, the effect of TLR4 activation on cell proliferation is something more controversial. While some studies reported that TLR4 did not affect cell proliferation (Kadono et al., 1999; Huang et al., 2014; Albiero et al., 2015; Li et al., 2016), others revealed opposite results (Wang et al., 2009; He et al., 2015). These discrepancies might be related to the specific agonist used to activate the receptor (DAMPs or PAMPs). Also, some divergent outcomes in terms of proliferation upon TLR4 activation might be attributable to the dissimilar expression of TLR4 across the investigated cell types (Tomic et al., 2011). In addition, the different effect of TLR4 activation on cell proliferation might be due to the variation in TLR4 agonists concentrations (Wang et al., 2009). Supporting this idea Wang et al. (2009) described that lower LPS concentrations promoted MSCs proliferation, while higher LPS concentration exhibited the opposite effect.

## TLR4 Effect on Osteoblast Mediated Osteoclastogenesis

The osteoclast is a multinucleated cell type formed by the fusion of monocytes/macrophages. Osteoclasts main function is the resorption of the bone, a process that mainly consists of the digestion of the mineral and organic matrix of the bone (Indo et al., 2013). The receptor activator of nuclear factor kappa-B ligand (RANKL), a cytokine of the TNF family, is a key factor in this process. RANKL in the presence of the macrophage-colony stimulating factor (M-CSF) boost the osteoclast generation (Indo et al., 2013; Charles and Aliprantis, 2014). Conversely, osteoprotegerin (OPG), a decoy receptor for RANKL, is a factor that blocks this process (Lacey et al., 2012; Charles and Aliprantis, 2014).

**FIGURE 4:**
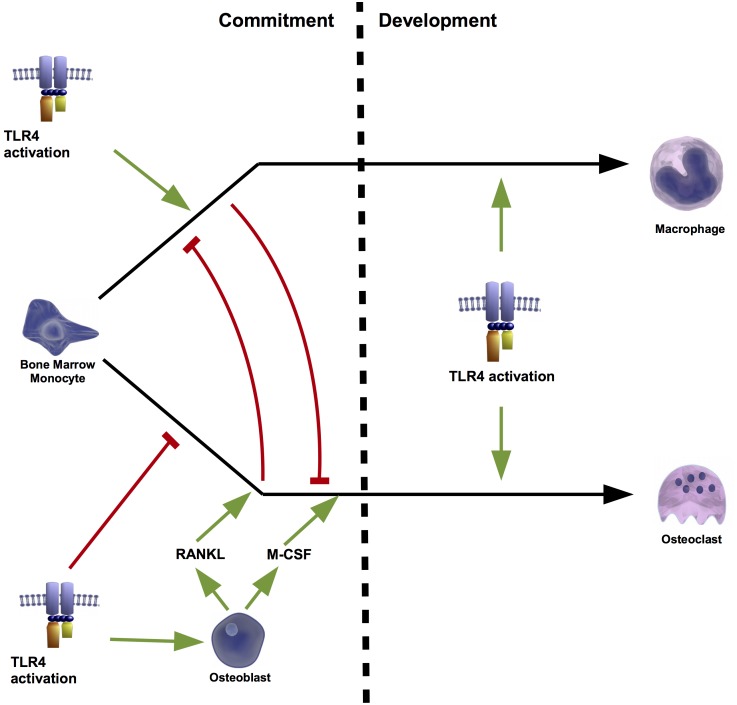
Osteoclastogenesis regulated by osteoblasts. TLR4 plays a key role in the cell fate of bone marrow monocytes (BMMs). During the first stage of commitment, TLR4 activation alone, promotes the conversion of these precursor cells into macrophages, and blocks osteoclastogenesis. However, osteoblasts can shift this fate to the formation of osteoclasts. TLR4 activation of osteoblasts induces the production of receptor activator of nuclear factor kappa-B ligand (RANKL) and macrophage-colony stimulating factors (M-CSF). The presence of these factors during the commitment phase prevents the macrophage fate and drives to osteoclast formation. Instead during the development stage, TLR4 activation triggers both cell fates. Thus, osteoclasts formation mediated by TLR4 depends on the presence or absence of osteoblast-derived RANKL during the commitment stage.

*In vitro* and *in vivo* experiments have extensively associated TLR4 agonism to the stimulation of the osteoclastogenesis (Sismey-Durrant and Hopps, 1987; Orcel et al., 1993; Hayashi et al., 2004). Accordingly, it was demonstrated in C3H/HeJ mice, which have a mutated TLR4, that activation of this receptor is associated to bone resorption (Nakamura et al., 2008). Moreover, TLR4 activation has also been related with several activities involved in osteoblast-mediated osteoclastogenesis (Shi et al., 2006). In fact, its activation in mouse osteoblasts induced the expression of RANKL mRNA (Kikuchi et al., 2001; Zou et al., 2003; Tang et al., 2011) and protein (Tang et al., 2011) without modifying the expression of OPG (Kikuchi et al., 2001; Zou et al., 2003). This induction was mediated by the extracellular signal-regulated kinase (ERK) (Kikuchi et al., 2001), the c-Jun N-terminal kinase (JNK) (Tang et al., 2011), and the PKC (Kikuchi et al., 2001). Moreover RANKL induction was independent of other inflammatory factors associated to TLR4 activation, such as TNFα and the PGE2 (Kikuchi et al., 2001). Together with RANKL up-regulation, TLR4 activation in mouse osteoblasts also induced the expression of M-CSF (Zou et al., 2003), which supported that TLR4 activation in osteoblasts may contribute to bone resorption (Kikuchi et al., 2001). In line with this, coculture of mouse primary osteoblast and hematopoietic cells in the presence of a TLR4 agonist stimulated the formation of osteoclasts (Yang et al., 2005). The effect of TLR4 activation on the osteoclastogenesis was attributed to osteoblasts TLR4 activation (Zhuang et al., 2007) because its activation in mouse bone marrow monocytes (BMMs) (osteoclast precursors) without co-culturing with osteoblasts failed to promote the formation of osteoclasts (Zhuang et al., 2007; Liu et al., 2009). Liu et al further observed that TLR4 activation inhibited osteoclastogenesis from mouse BMM but stimulated from those pre-treated with RANKL or co-cultured with osteoblasts (Liu et al., 2009). Interestingly these authors found that RANKL-mediated BMM commitment to osteoclasts was a prerequisite for TLR4-induced osteoclastogenesis (Liu et al., 2009). Conversely, the priming of mouse BMM by TLR4 activation blocked RANKL-mediated osteoclastogenesis (Liu et al., 2009) (**Figure 4**).

## TLR4 Effect on Osteoblast Differentiation and Metabolism

Several reports have described anabolic properties of TLR4 activation on osteoblast (Mo et al., 2008; van den Berk et al., 2009; He et al., 2015; Ma et al., 2017). This activation in human primary osteoblasts up-regulated the expression of key osteoblastic markers (Ma et al., 2017). It was also observed that prolonged TLR4 activation of human MSCs up-regulated their osteoblastic differentiation without affecting their proliferation rate (Mo et al., 2008). However, this long-term activation of TLR4 was also related to a reduction in their expression of TLR4 (Mo et al., 2008). Nonetheless, other reports in human UCB MSCs (van den Berk et al., 2009), human periodontal ligament MSCs (Albiero et al., 2015), adipose tissue MSCs (Raicevic et al., 2012), and bone marrow MSCs (Raicevic et al., 2012) revealed that TLR4 activation enhanced their osteoblastic differentiation (van den Berk et al., 2009; Raicevic et al., 2012). Likewise, in mouse MSCs TLR4 activation also promoted their osteoblastic differentiation as well as its proliferation (He et al., 2015). In these cells TLR4 activation up-regulated wingless-INT (Wnt) family member 3A (Wnt3a) and Wnt family member 5A (Wnt5a), two major factors involved in the commitment of MSCs toward the osteoblastic cell fate (He et al., 2015). siRNA inhibition of these factors blocked the proliferative and osteoblastogenic activities of TLR4 activation suggesting that wingless-INT (Wnt) signaling was the driving force underlying to the TLR4 activation (He et al., 2015). According to this MSCs from MyD88^-/-^ mice lacked the capacity to differentiate into osteoblasts cells (Pevsner-Fischer et al., 2007), which suggested that TLR4 associated MyD88-dependent signaling pathway was required to achieve the osteoblastic phenotype (Pevsner-Fischer et al., 2007).

In contrast to these data attributing an anabolic or pro-osteoblastogenic role to TLR4 activation, other authors observed that bone healing was accelerated in TLR4^-/-^ mice after a skull lesion (Wang et al., 2017). However, a similar effect was also observed in a myeloid cell-specific TLR4 knockout mouse, which suggested that the anabolic activities observed in the TLR4^-/-^ mice were not related to the absence of TLR4 in the osteoblasts (Wang et al., 2017). Martino et al. (2016) did not find the same effect in different TLR4^-/-^ mice. Nonetheless, since IL1R and TLR4 share their MyD88-dependent signaling pathway, it is of interest that the same authors also found that MyD88 deficient mice, as well as IL1R^-/-^ mice, exhibited a faster bone regeneration than their wild type littermates (Martino et al., 2016). Likewise, MyD88 deficient mice were resistant to PAMP-induced bone loss (Madeira et al., 2013), which was associated to less osteoclast formation as well as increased expression of osteoblastic markers (Madeira et al., 2013). The TLR4/MyD88-independent signaling pathway, which involves the signaling through TRIF, was not involved in the TLR4-mediated bone loss since TRIF^-/-^ mice were not resistant PAMP-induced bone loss (Madeira et al., 2013). This was consistent with other reports about TRIF-independent inflammatory responses elicited by TLR4 activation in mouse osteoblasts (Sato et al., 2004). According to the TLR4-mediated inhibition of certain anabolic processes, TLR4 activation in differentiating mouse primary osteoblasts (Bandow et al., 2010), mouse osteoblastic cell line MC3T3-E1 (Liu et al., 2016), or in mouse MSCs (Chen et al., 2014) inhibited the matrix mineralization (Bandow et al., 2010), whereas this activation did not inhibit the matrix mineralization of mouse MyD88^-/-^-derived primary osteoblasts (Bandow et al., 2010). Moreover, unlike occurred in MyD88^-/-^ osteoblasts, TLR4 activation of wild type osteoblasts up-regulated the mRNA of the activating transcription factor 4 (ATF4), as well as down-regulated osteoblastic transcription factors like the runt related transcription factor 2 (Runx2), and osterix (Sp7) (Bandow et al., 2010). Similar results were observed in primary rat calvaria osteoblasts (Kadono et al., 1999). In these cells TLR4 activation inhibited the expression of different osteoblastic markers, including alkaline phosphatase (ALP) osteocalcin, and osteopontin (Kadono et al., 1999). In the same way TLR4 activation in the osteoblast cell line MC3T3-E1 inhibited ALP activity and the mRNA expression of ALP, osteocalcin, and Runx2 (Liu et al., 2016). Interestingly, it was depicted that the inhibitory effect of TLR4 activation on mouse MSCs osteoblastic differentiation was mediated by the inhibitory crosstalk of the TLR4/MyD88/NFκB pathway over the anabolic BMP/Smad pathway (Huang et al., 2014).

Experiments performed in human primary osteoblasts (Muthukuru and Darveau, 2014) with diverse PAMP preparations that presented different effects on TLR4 function revealed that while strong TLR4 agonism involved inhibition of the osteoblastic markers, weak TLR4 agonism or TLR4 antagonism up-regulated them (Muthukuru and Darveau, 2014). Interestingly, it was suggested that the effect of TLR4 activation or inhibition on the osteoblastic markers was associated to the inflammatory response elicited (Muthukuru and Darveau, 2014). According to this, strong TLR4 agonists at very low concentrations also exhibit an anabolic effect on the osteoblastic markers (Muthukuru and Darveau, 2014).

## Effect of TLR4 on Other Osteoblast Activities

Osteoblasts can also contribute to bone resorption through the production of key degradative proteinases such as the matrix metalloproteinases 13 (MMP13) (Gao et al., 2013). In this sense it is noteworthy that TLR4 activation also up-regulated the expression of this catabolic factor (Gao et al., 2013).

Hyperlipidemic or hyperglycemic environments can alter osteoblasts and bone metabolism. TLR4 activation has been related to these alterations (Moriya et al., 2014; Rendina-Ruedy et al., 2016). Specifically, in rat osteoblasts TLR4 activation upon stimulation with palmitate, a hyperlipidemic environment, induced the secretion of the vascular endothelial growth factor 120 (VEGF120) (Moriya et al., 2014), which has been related with an abnormal bone metabolism. Moreover in a mouse animal model of high-fat diet-induced glucose intolerance TLR4 defective signaling (C3H/HeJ mice) was associated to a later onset and reduced bone alterations (Rendina-Ruedy et al., 2016).

In contrast to this, in co-culture experiments performed with human osteoblasts and endothelial cells it was observed that TLR4 activation enhanced angiogenesis (Ma et al., 2017), which is a key component of bone repair (Hankenson et al., 2011).

Osteoblast migration is a key process in skeletal development as well as in bone regeneration (Li et al., 2016). TLR4 activation in mouse MSCs inhibited the migration ability of these cells (Chen et al., 2014). However, other authors observed in primary rat osteoblasts that TLR4 activation by the DAMP HMGB1 promoted their migration (Li et al., 2016). Nonetheless, in the mouse osteoblastic cell line MC3T3-E1 this effect of HMGB1 was not attributed to TLR4 activation (Qiu et al., 2016).

## Future Directions

Inflammation regulation is required to achieve a healthy bone metabolism. Excessive inflammation or inhibition of inflammatory responses have been linked to bone resorption and altered bone fracture healing. Therefore, future research should be focused on the characterization of the magnitude and duration of TLR4 agonism associated to these bone alterations. Likewise, as it was established for osteoclasts, it would be necessary to address how TLR4 activation affects osteoblasts differentiation at each stage of the process. Moreover, considering that excessive TLR4 signaling implies bone destruction further studies aimed to investigate novel therapeutic weapons should be carry on.

## Conclusion

Inflammation plays a key role in bone metabolism. Bone inflammatory responses are partially meditated by PRRs such as TLR4. This receptor that recognizes DAMPS and PAMPS has been related with the onset and development of different musculoskeletal pathologies where bone physiology is altered. According to this, TLR4 is expressed in cells that directly participate in bone metabolism; namely osteoblasts, osteoclasts, and MSCs. Nonetheless, its expression is uneven across these cells. Despite this, TLR4 activation in osteoblasts and MSCs mediates a similar production of multiple cytokines, chemokines, and inflammatory mediators.

TLR4 signaling in osteoblasts and MSCs has also been involved in the regulation of cell viability and proliferation. However, these effects were not consistent across different studies. This might be explained by confounding factors. Some of these factors could also be responsible for the un-consistent effects of TLR4 activation on osteoblasts differentiation, where either anabolic or catabolic effects have been reported.

It is well-known that osteoblasts contribute to osteoclastogenesis. Interestingly, activation of TLR4 in osteoblasts promotes this process despite that its activation in osteoclast precursor cells inhibits their commitment.

Altogether, these data suggest that TLR4 might be a potential target to modulate bone metabolism. However, further studies are required to elucidate the precise role of this receptor on osteoblasts.

## Author Contributions

AA-P, EF-T, and RG: study design and conception. AA-P, EF-T, MG-F, AJ-M, VL, JP, OG, and RG: declare that they collaborate in the search and selection of the papers, as in manuscript drafting. All of them have seen and approved the final version.

## Conflict of Interest Statement

The authors declare that the research was conducted in the absence of any commercial or financial relationships that could be construed as a potential conflict of interest.
